# The calcium‐dependent protein kinase (CDPK) and CDPK‐related kinase gene families in *Hevea brasiliensis*—comparison with five other plant species in structure, evolution, and expression

**DOI:** 10.1002/2211-5463.12163

**Published:** 2016-12-12

**Authors:** Xiao‐Hu Xiao, Meng Yang, Jin‐Lei Sui, Ji‐Yan Qi, Yong‐Jun Fang, Song‐Nian Hu, Chao‐Rong Tang

**Affiliations:** ^1^ Key Lab of Rubber Biology, Ministry of Agriculture & Rubber Research InstituteChinese Academy of Tropical Agricultural SciencesDanzhouHainanChina; ^2^ Beijing Institute of GenomicsChinese Academy of SciencesBeijingChina; ^3^ College of AgricultureHainan UniversityHaikouHainanChina

**Keywords:** calcium‐dependent protein kinase, CDPK‐related kinase, gene expression, *Hevea brasiliensis*, structure and evolution

## Abstract

Calcium‐dependent protein kinases (CDPKs or CPKs) play important roles in various physiological processes of plants, including growth and development, stress responses and hormone signaling. Although the CDPK gene family has been characterized in several model plants, little is known about this gene family in *Hevea brasiliensis* (the Para rubber tree). Here, we characterize the entire *H. brasiliensis* CDPK and CDPK‐related kinase (CRK) gene families comprising 30 *CDPK* genes (*HbCPK1* to *30*) and nine *CRK* genes (*HbCRK1* to *9*). Structure and phylogeny analyses of these *CDPK* and *CRK* genes demonstrate evolutionary conservation in these gene families across *H. brasiliensis* and other plant species. The expression of *HbCPK* and *HbCRK* genes was investigated via Solexa sequencing in a range of experimental conditions (different tissues, phases of leaf development, ethylene treatment, and various abiotic stresses). The results suggest that *HbCPK* and *HbCRK* genes are important components in growth, development, and stress responses of *H. brasiliensis*. Parallel studies on the *CDPK* and *CRK* gene families were also extended to five other plant species (*Arabidopsis thaliana*,* Oryza sativa*,* Populus trichocarpa*,* Manihot esculenta,* and *Ricinus communis*). The *CDPK* and *CRK* genes from different plant species that exhibit similar expression patterns tend to cluster together, suggesting a coevolution of gene structure and expression behavior in higher plants. The results serve as a foundation to further functional studies of these gene families in *H. brasiliensis* as well as in the whole plant kingdom.

**Abbreviations**
CDPK/CPK: calcium‐dependent protein kinase
CRK: CDPK‐related kinase
QPCR: real‐time quantitative reverse transcription polymerase chain reaction
SRA: Sequence Read Archive

Plants have evolved a series of survival mechanisms to adapt to diverse environmental challenges, including drought, salinity, wounding, and low temperatures 1, 2, 3, 4. Calcium (Ca^2+^), functioning as a second messenger of plant cells, plays an essential role in various signaling transduction pathways 5, 6. The changes in Ca^2+^ concentration are sensed by several Ca^2+^ sensors or Ca^2+^‐binding proteins. To date, three major classes of Ca^2+^‐binding proteins, including calcium‐dependent protein kinases (CDPKs or CPKs), calmodulins (CaMs), and CaM‐like proteins (CMLs) and calcineurin B‐like proteins (CBLs), have been characterized in higher plants 7, 8. The CDPKs constitute one of the largest calcium‐sensing subfamilies of serine/threonine protein kinases that have been identified throughout the plant kingdom, from algae to angiosperms as well as in some protozoans, but not in animals 9. The CDPK proteins have four characterized domains: an N‐terminal variable region, a Ser/Thr kinase catalytic domain, an autoregulatory/autoinhibitory domain, and a calmodulin‐like domain containing EF‐hands for Ca^2+^ binding 10, 11, 12.

Accumulating evidence indicates that CDPKs participate in plant responses to a variety of abiotic and biotic stresses, as well as in plant development 13, 14, 15. In *Arabidopsis thaliana*,* CPK10* participates in abscisic acid (ABA) and Ca^2+^‐mediated stomatal regulation in response to drought stress 16. Two homologs, *AtCPK4* and *AtCPK11*, acting as positive regulators in ABA signaling pathways, are involved in seed germination, seedling growth, stomatal movement, and salt stress tolerance 17. Another *CDPK* gene, *AtCPK12*, has been characterized as a negative ABA‐signaling pathway regulator 18, while *AtCPK6* demonstrates crucial roles in responses to drought and salt stresses 19. In *Oryza sativa*, transgenic plants overexpressing *OsCDPK7*/*OsCPK13* show enhanced resistance to cold, drought, and salt stress 20. *OsCDPK13*/*OsCPK7* and *OsCPK21* are involved in responses to cold and salt stress, respectively 21, 22. In *Nicotiana tabacum*, two *CDPK* genes, *NtCDPK2* and *NtCDPK3*, play an essential role in the defense response, and *NtCDPK2* functions together with the stress‐induced MAPKs (mitogen‐activated protein kinases) to control response specificity to abiotic and biotic stresses 23, 24. In addition, genome‐wide expression patterns of *CDPK* genes have been characterized in *O. sativa*,* Zea mays*,* Populus trichocarpa*, and *Brassica napus*, and they point to important roles in the regulation of abiotic stresses, hormones, and the intrinsic developmental program in plant growth and development 25, 26, 27, 28.

Natural rubber (*cis*‐1,4‐polyisoprene, NR) is an important industrial raw material, and the sole commercial source of NR is *Hevea brasiliensis* (the Para rubber tree), a perennial tropical tree species 29. NR is synthesized and stored in the laticifer cells, which are differentiated from the cambium and arranged in concentric rings in the phloem region 29, 30. The bark of rubber tree is incised every 2–3 days to sever the laticifer rings and collect the latex, and this process is called tapping 29. At each tapping, several tens to a few hundred milliliters of latex per tree are expelled from the laticifers and harvested for sustainable rubber production. Application of ethylene gas or ethephon (2‐chloroethylphosphonic acid, an ethylene generator) to the trunk bark of the rubber tree increases rubber yield. However, the underlying mechanisms in ethylene stimulation are yet to be elucidated, although ethylene signaling and response are assumed to play critical roles 29. In a previous report, one *CDPK* gene (*HbCDPK1*) was shown to be induced by ethephon in *H. brasiliensis* 31. Both environmental and harvesting stresses affect rubber yield. For example in tapping, the moderate stresses generate a positive stimulatory effect on latex production in virgin (previously untapped) rubber trees, bringing the rubber yield from a meager exudation at the first tapping to more than a hundred milliliters after 7–10 consecutive tappings 32. However, excessive environmental and harvesting stresses, especially a combination of overtapping and overstimulation by ethylene or ethephon, lead to the occurrence of tapping panel dryness (TPD), a physiological disorder that can result in complete stoppage of latex flow in the rubber tree 29. In view of the importance of *CDPK* genes in stress responses and hormone signaling in other plants 6, it is worthwhile investigating the *CDPK* gene family in the rubber tree in relation to the regulation of latex production.

In this study, we performed a genome‐wide analysis of the *CDPK* and *CRK* gene families in *H. brasiliensis* and compared the results with those from five other plant species, i.e. *Manihot esculenta*,* Ricinus communis, A. thaliana*,* O. sativa,* and *P. trichocarpa*. The study encompassed a total of 161 *CDPK* and 45 *CDPK*‐related kinase (*CRK*) genes, the expression patterns of which were analyzed in different plant tissues in response to various treatments, and at several phases of tissue development. In addition, the gene structure and phylogeny of these genes were also compared, and all the results obtained will help further understanding of the roles of *CDPK* and *CRK* genes in the regulation of latex production and regeneration. Data on *CDPK* and *CRK* genes and their expression in six plant species would also contribute to the understanding of the structure of these gene families and the functions of their members in the plant kingdom.

## Results and Discussion

### Genome‐wide identification of CDPK and CRK gene families in *H. brasiliensis* and five other plant species

We identified all *CDPK* and *CRK* gene family members in six plant species (*H. brasiliensis*,* A. thaliana*,* O. sativa*,* P. trichocarpa*,* M. esculenta*, and *R. communis*) from their published genome sequences 33, 34, 35, 36, 37, 38, 39. The latest genome and protein sequences of these species were downloaded from Phytozome v10. Local blast searches of the genomes were performed by using the published *CDPK* and *CRK* sequences of three model plants of *A. thaliana*,* O. sativa,* and *P. trichocarpa* as queries 12, 26, 28, 40. This analysis identified a total of 161 *CDPK* genes and 45 *CRK* genes in the six plant species, including 39 *H. brasiliensis CDPK* and *CRK* genes (*HbCPK1* to *30,* and *HbCRK1* to *9*, Table 1a), 31 *M. esculenta* genes (*MeCPK1* to *22*, and *MeCRK1* to *9*, Table 1b), 21 *R. communis* genes (*RcCPK1* to *16,* and *RcCRK1 to 5*, Table 1c), 42 *A. thaliana* genes (*AtCPK1* to *34,* and *AtCRK1* to *8,* Table S1–4), 39 *P. trichocarpa* genes (*PtCDPK1 to 30,* and *PtCRK1* to *9*, Table S1–5), and 34 *O. sativa* genes (*OsCPK1* to *29,* and *OsCRK1* to *5*, Table S1–6). The gene numbers of *CDPK* and *CRK* families identified here for the three model plants agree well with those previously reported 12, 28, 41.

**Table 1:** Characteristics of *CDPK* and *CRK* genes in three *Euphorbiaceae* members, *Hevea brasiliensis, M. esculenta,* and *R. communis*

a) *H. brasiliensis*									
*HbCDPKs* or *HbCRKs*	ID	CDS length in bp	Predicted protein			No. of introns	Group		
			Length (aa)	Isoelectric point	Mol Wt				
*HbCPK1*	387072309	1764	588	5.22	65750.55	6	CPKI		
*HbCPK2*	–	1602	534	5.4	59450.32	7	CPKII		
*HbCPK3*	–	1704	568	6.29	64610.86	6	CPKIII		
*HbCPK4*	387060177	1602	534	6.75	60132.81	7	CPKII		
*HbCPK5*	387068553	1626	542	5.86	61341.08	6	CPKI		
*HbCPK6*	387057753	1722	574	5.14	63872.63	6	CPKI		
*HbCPK7*	–	1569	523	5.47	58217	7	CPKII		
*HbCPK8*	–	1647	549	6.73	62648.76	6	CPKIII		
*HbCPK9*	387061935	1590	530	6.13	59626.89	8	CPKII		
*HbCPK10*	–	1305	435	5.17	49186.02	9	CPKIII		
*HbCPK11*	387060219	1602	534	6.07	60196.74	7	CPKII		
*HbCPK12*	387067377	1500	500	5.54	56598.5	6	CPKI		
*HbCPK13*	387058668	1593	531	6.25	60383.85	7	CPKIII		
*HbCPK14*	–	1584	528	6.11	59945.36	7	CPKII		
*HbCPK15*	387059122	1683	561	5.54	62869.32	6	CPKI		
*HbCPK16*	387063507	1551	517	5.36	58166.27	6	CPKI		
*HbCPK17*	–	1590	530	6.09	59573.92	8	CPKII		
*HbCPK18*	–	1581	527	5.91	59425.87	6	CPKIII		
*HbCPK19*	387062498	1581	527	5.86	59368.88	6	CPKIII		
*HbCPK20*	387059814	1695	565	8.97	63976.74	11	CPKIV		
*HbCPK21*	387063125	1695	565	9.25	64073.67	11	CPKIV		
*HbCPK22*	–	1653	551	5.68	62621.56	7	CPKIII		
*HbCPK23*	387062143	1506	502	5.38	56753.76	6	CPKI		
*HbCPK24*	–	1752	584	5.44	65647.88	6	CPKI		
*HbCPK25*	–	1770	590	5.07	65770.61	6	CPKI		
*HbCPK26*	–	1599	533	5.98	60567.48	7	CPKIII		
*HbCPK27*	387059234	1683	561	5.62	62799.22	6	CPKI		
*HbCPK28*	–	1893	631	5.47	70785.55	6	CPKI		
*HbCPK29*	–	1602	534	6.49	60629.41	7	CPKIII		
*HbCPK30*	EU581818	1671	556	5.15	66350.8	7	CPKIII		
*HbCRK1*	387066783	1890	630	8.57	70199.19	10	CRK		
*HbCRK2*	–	1875	625	8.69	69866.87	10	CRK		
*HbCRK3*	387056042	1728	576	8.56	64518.92	10	CRK		
*HbCRK4*	–	1803	601	9.03	66945.41	10	CRK		
*HbCRK5*	–	1806	602	8.89	67117.41	10	CRK		
*HbCRK6*	–	1713	571	8.53	63969.39	10	CRK		
*HbCRK7*	–	1749	583	7.61	65483.01	10	CRK		
*HbCRK8*	387060548	1782	594	8.99	66942.73	10	CRK		
*HbCRK9*	–	1782	594	8.81	66864.31	10	CRK		

b) *M. esculenta*									
MeCPKs or MeCRKs	PACID	Chromosome /scaffold	Coordinates (5′–3′)	CDS length in bp	Predicted protein			No. of introns	Group
					Length (aa)	Isoelectric point	Mol Wt		
*MeCPK1*	17994103	scaffold08542	688509–691868	1932	644	5.02	72133.41	6	CPKI
*MeCPK2*	17976152	scaffold12865	417463–425519	1722	574	5.08	64055.78	6	CPKI
*MeCPK3*	17971973	scaffold06582	497705–502368	1674	558	5.52	62573.99	6	CPKI
*MeCPK4*	17984594	scaffold02040	210153–214213	1674	558	5.53	62874.42	6	CPKI
*MeCPK5*	17969086	scaffold08359	2363266–2367666	1647	549	6.57	62586.62	6	CPKIII
*MeCPK6*	17971267	scaffold10963	249788–253275	1614	538	6.12	60531.19	7	CPKIII
*MeCPK7*	17968159	scaffold12121	105306–108270	1611	537	5.91	61183.21	8	CPKIII
*MeCPK8*	17966798	scaffold04681	369819–373312	1605	535	6.43	60124.83	7	CPKIII
*MeCPK9*	17975050	scaffold06278	662521–666044	1602	534	6.2	60172.73	7	CPKII
*MeCPK10*	17971020	scaffold05280	746256–751592	1596	532	6.05	59686.91	8	CPKII
*MeCPK11*	17987801	scaffold03241	233760–237133	1596	532	6.69	59901.47	7	CPKII
*MeCPK12*	17980235	scaffold04075	9032–12714	1593	531	7	60388.97	7	CPKIII
*MeCPK13*	17980210	scaffold02242	186503–191550	1593	531	5.85	60293.62	7	CPKIII
*MeCPK14*	17981799	scaffold06916	982685–986060	1587	529	5.24	58756.69	8	CPKII
*MeCPK15*	17967725	scaffold07991	65801–68074	1584	528	5.42	58744.47	7	CPKII
*MeCPK16*	17990251	scaffold06754	43023–46027	1584	528	5.53	60260.13	7	CPKIII
*MeCPK17*	17988164	scaffold06814	209569–213530	1533	511	5.61	57584	6	CPKI
*MeCPK18*	17967898	scaffold00853	48033–52009	1509	503	5.33	56559.32	6	CPKI
*MeCPK19*	17992600	scaffold02811	52985–57215	1506	502	5.4	56697.66	6	CPKI
*MeCPK20*	17971395	scaffold00467	131837–136151	1503	501	5.49	56753.78	6	CPKI
*MeCPK21*	17979246	scaffold07520	2142987–2147997	1746	582	8.97	66122.79	11	CPKIV
*MeCPK22*	17966476	scaffold06512	925585–931028	1704	568	8.99	64446.09	11	CPKIV
*MeCRK1*	17992954	scaffold03060	94515–100498	1890	630	8.81	70469.23	10	CRK
*MeCRK2*	17982960	scaffold08352	120824–127160	1890	630	8.89	70275.35	10	CRK
*MeCRK3*	17975749	scaffold03581	287483–294244	1809	603	9.02	67415.87	10	CRK
*MeCRK4*	17972581	scaffold03846	35776–42796	1809	603	8.96	67318.61	10	CRK
*MeCRK5*	17983969	scaffold06353	3656–9704	1794	598	7.58	67487.91	12	CRK
*MeCRK6*	17977726	scaffold11584	15400–22546	1773	591	9.06	66584.36	10	CRK
*MeCRK7*	17988236	scaffold02895	195081–201488	1752	584	8.61	65642.26	10	CRK
*MeCRK8*	17983077	scaffold04094	24034–29752	1728	576	8.23	64515.79	10	CRK
*MeCRK9*	17961210	scaffold09761	236897–241551	1290	430	6.61	48548.92	10	CRK

c) *R. communis*									
RcCPKs or RcCRKs	PACIDa	Chromosome /scaffold	Coordinates (5′–3′)	CDS length in bp	Predicted protein			No. of introns	Group
					Length (aa)	Isoelectric point	Mol Wt		
*RcCPK1*	16802692	29333	292448–295456	1923	641	5.98	72059.09	6	CPKI
*RcCPK2*	16802702	29333	380788–386245	1752	584	5.32	65395.33	6	CPKI
*RcCPK3*	16811562	29852	749078–754396	1734	578	5.39	64238.02	6	CPKI
*RcCPK4*	16817768	30100	635436–641118	1683	561	5.46	62880.09	6	CPKI
*RcCPK5*	16810728	29842	470020–473807	1653	551	6.2	62094.68	7	CPKII
*RcCPK6*	16820071	30147	4261059–4265373	1647	549	6.4	62372.39	6	CPKIII
*RcCPK7*	16819478	30142	206467–209308	1608	536	5.69	59521.44	7	CPKII
*RcCPK8*	16821242	30169	1209760–1213471	1608	536	6.49	60331.98	7	CPKIII
*RcCPK9*	16799163	27777	46786–50324	1599	533	6.28	60220.61	7	CPKII
*RcCPK10*	16812395	29896	35082–37969	1599	533	5.82	60477.19	7	CPKIII
*RcCPK11*	16808108	29761	124817–128087	1587	529	6.65	60101.64	7	CPKIII
*RcCPK12*	16822115	30170	3717810–3721220	1587	529	6.21	59735.14	7	CPKII
*RcCPK13*	16823697	30190	3182769–3187309	1584	528	6.09	59244.51	8	CPKII
*RcCPK14*	16800511	28308	38002–44802	1575	525	5.82	59384.75	6	CPKIII
*RcCPK15*	16806882	29728	126431–131585	1491	497	5.33	56030.04	6	CPKI
*RcCPK16*	16810129	29830	39047–45797	1725	575	9.04	65179.79	11	CPKIV
*RcCRK1*	16799638	27964	46400–53108	1806	602	8.96	67299.67	10	CRK
*RcCRK2*	16808369	29780	205986–212347	1794	598	9.15	67650.44	10	CRK
*RcCRK3*	16817534	30089	1472595–1478763	1767	589	8.98	66035.89	10	CRK
*RcCRK4*	16805369	29676	110174–114278	1401	467	7.67	51967.11	7	CRK
*RcCRK5*	16807073	29733	60296–65221	1536	512	7.32	57231.11	10	CRK

All the 161 CDPKs share conserved CDPK domains, including an N‐terminal variable domain, a protein kinase domain, an autoinhibitory domain, and Ca^2+^‐binding EF‐hands. While CRKs share degenerative Ca^2+^‐binding EF‐hands compared to CDPKs. The lengths of the CDPK‐ and CRK‐coding regions (CDS) were similar among the six plant species examined, ranging from 1305 to 1893 bp in *H. brasiliensis*, 1452 to 1938 bp in *A. thaliana*, 1359 to 1896 bp in *O. sativa,* 1431 to 1839 bp in *P. trichocarpa*, 1290 to 1932 bp in *M. esculenta*, and 1401 to 1923 bp in *R. communis* (Table 1, Table S1). The molecular weights of the predicted 206 CDPK and CRK proteins range from 48.5 to 72.3 kDa, while their isoelectric points (pI) fall between 5.02 and 9.36 (Table S1).

### Phylogenetic analysis of the CDPK and CRK gene families

**Figure 1:**
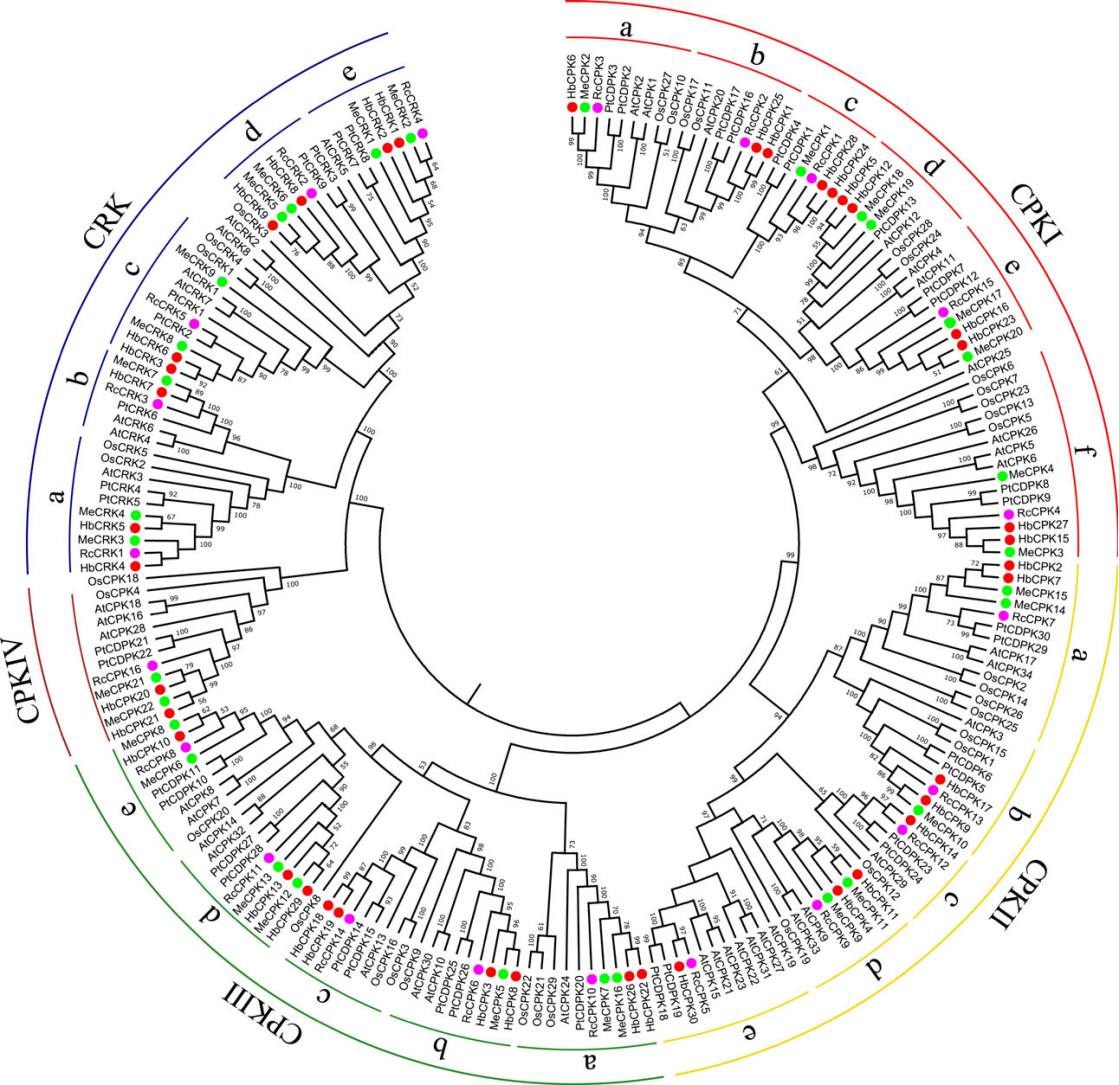
Phylogenetic analysis of the *CDPK* and *CRK* genes in *H. brasiliensis* and five other plant species. An unrooted phylogenetic tree of plant CDPK and CRK proteins was constructed using the NJ method with the MEGA 6.0 program. Plant species and their CDPK and CRK proteins are: *H. brasiliensis*, HbCPK1 to 30 and HbCRK1 to 9, marked with red dots; *A. thaliana*, AtCPK1 to 34 and AtCRK1 to 8; *P. trichocarpa*, PtCDPK1 to 30 and PtCRK1 to 9; *O. sativa*, OsCPK1 to 29 and OsCRK1 to 5; *M. esculenta*, MeCPK1 to 22 and MeCRK1 to 9, marked with green dots; *R. communis*, RcCPK1 to 16 and RcCRK1 to 5, marked with pink dots.

In order to establish the phylogenetic relationships in the *CDPK* and *CRK* gene families among *H. brasiliensis* and the five other plant species, we aligned the 206 plant CDPK and CRK protein sequences and constructed a phylogenetic tree as shown in Fig. 1. The plant CDPK and CRK proteins that clustered into five major groups with high bootstrap values are named CPKI, CPKII, CPKIII, CPKIV, and CRK. The proteins in three CPK groups (I–III) and the CRK group are further classified into a number of distinct subgroups consisting of both dicot and monocot proteins. The 39 *H. brasiliensis* genes are scattered among the five groups (CPKI, CPKII, CPKIII, CPKIV, and CRK) with 11, 8, 9, 2, and 9 isoforms, respectively. Similarly, the *CDPK* and *CRK* family genes in the five other species are also clustered into different groups (CRKI, II, III, and IV, and CRK): 10, 13, 8, 3, and 8 isoforms, respectively, in *A. thaliana*; 11, 8, 8, 2, and 5 in *O. sativa*; 11, 8, 9, 2, and 9 in *P. trichocarpa*; 8, 5, 7, 2, and 9 in *M. esculenta*; 5, 5, 5, 1, and 5 in *R. communis* (Table S1, Fig. 1). Unlike the five other plant species, *A. thaliana* has a smaller number of genes in the CPK I group than in the CPK II group, which is apparently due to the striking expansion of the subgroup of CPK IIe in *A. thaliana* (Fig. 1). In all six plant species, CPK IV has the smallest number of genes among the five CPK and CRK groups, and is most closely related to the CRK group rather than the other three CPK groups in the phylogenetic tree (Fig. 1), suggesting the divergence of CPK IV and CRK from a common ancestor comparatively recently. Generally, the HbCPK isoforms are more closely related to their putative CDPK orthologs in *M. esculenta* and *R. communis*, which are in the same family of *Euphorbiaceae* with *H. brasiliensis*, reflecting consistency in the evolution of CDPK isoforms and plant lineages.

**Figure 2:**
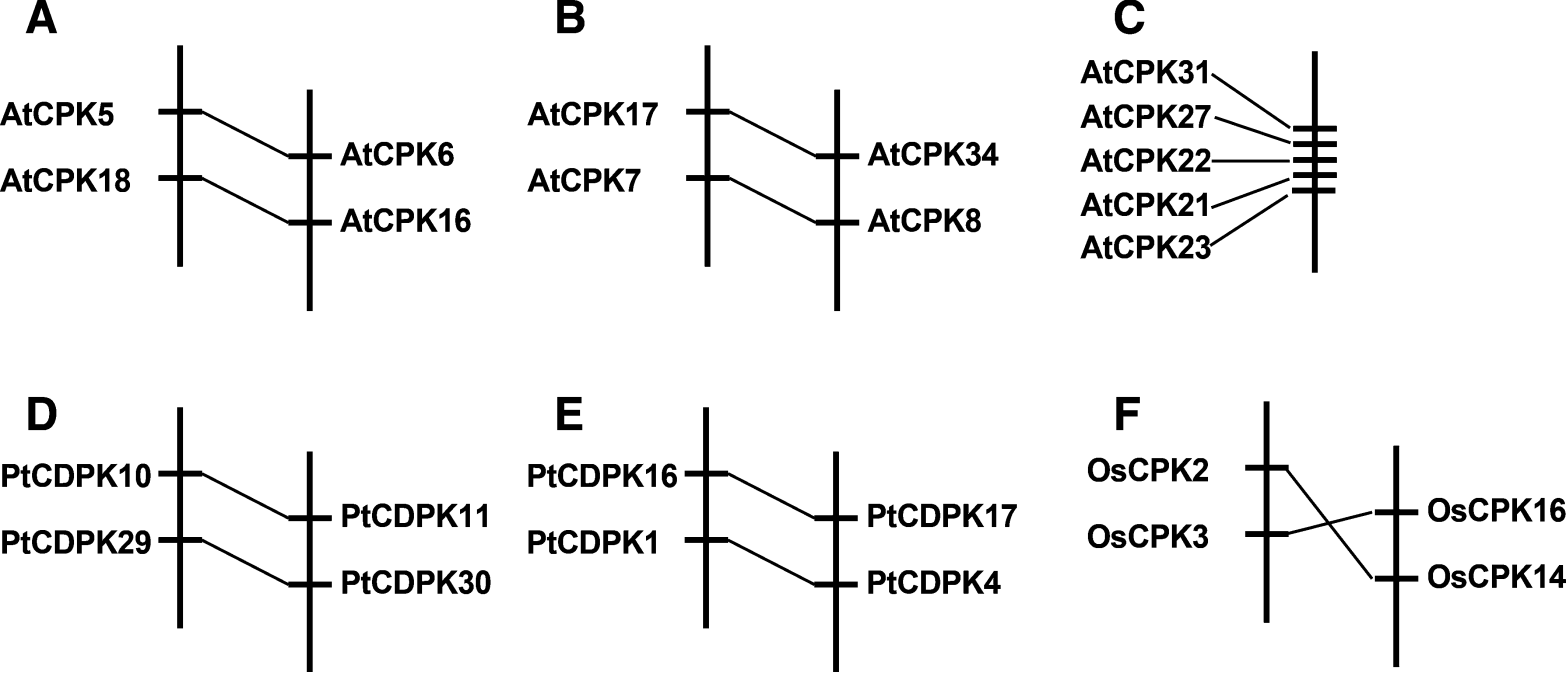
Chromosomal locations and segmental duplication events of *CDPK* genes in *A. thaliana, P. trichocarpa*, and *O. Sativa*. In *A. thaliana*, the paralogous gene pairs of *AtCPK5/6* and *AtCPK18/16* are located on Chromosome IV and II (A), *AtCPK17/34* and *AtCPK7/8* on Chromosome V (B), and the paralogous *AtCPK* gene cluster on Chromosome IV (C). In *P. trichocarpa*, the paralogous gene pairs of *PtCPK10/11* and *PtCPK29/30* are located on Chromosome I and IX (D)*,* and *PtCPK16/17* and *PtCPK1/4* on Chromosome VI and XVI (E). In *O. sativa*, the paralogous gene pairs of *OsCPK2/14* and *PtCPK3/16* are located on Chromosome I and V (F).

Phylogenetic analysis as well as amino acid sequence comparison revealed that with the exception of *R. communis*, universal existence of paralogous *CDPK* and *CRK* gene pairs were observed in the five other species. In *H. brasiliensis*, six such *CDPK* gene pairs (*HbCPK1/25*,* HbCPK2/7*,* HbCPK5/12*,* HbCPK18/19, HbCPK22/26, HbCPK24/28*) and one *CRK* gene pair (*HbCRK3/6*) were identified. In *A. thaliana*, there were nine *CDPK* gene pairs (*AtCPK1/2*,* AtCPK4/11, AtCPK5/6, AtCPK17/34, AtCPK9/33, AtCPK10/30, AtCPK14/32, AtCPK7/8, AtCPK16/18*), three *CRK* gene pairs (*AtCRK4/6, AtCRK1/7* and *AtCRK2/8*), and one paralogous gene cluster in subgroup CPK IIe (*AtCPK27, 31, 22, 15, 23* and *21*). In *M. esculenta,* nine *CDPK* (*MeCPK17/20, MeCPK18/19, MeCPK3/4, MeCPK9/11, MeCPK14/15, MeCPK16/7, MeCPK12/13, MeCPK6/*8, and *MeCPK22/21*) and four *CRK* (*MeCRK3/4, MeCRK8/9*,* MeCRK5/6*, and *MeCRK1/2*) gene pairs were identified. However, in this species the two genes in each pair do not cluster together as closely as in the other plant species examined in this study (Fig. 1), suggesting an earlier divergence of the two genes. In *P. trichocarpa*, 14 *CDPK* and four *CRK* gene pairs were identified, and in *O. sativa*, 11 closely related *CPK* pairs and one *CRK* pair were identified, the results of which are consistent with those from previous studies 28, 41. Upon further examining the genomic locations, we found that some of the paralogous gene pairs were located adjacent to each other, such as *PtCDPK10/11* and *PtCDPK29/30*,* PtCDPK16/17* and *PtCDPK1/4*,* AtCPK5/6* and *AtCPK18/16*,* AtCPK17/34* and *AtCPK7/8*,* OsCPK2/3* and *OsCPK16/14* (Fig. 2A,B,D–F). These adjacent gene pairs might have evolved from a common ancestor that underwent tandem duplication prior to the segmental duplication. Interestingly, of the six genes in the paralogous cluster observed in *A. thaliana*, five are closely located in the same chromosome (Fig. 2C), and are apparently derived from tandem duplication events.

### Structural organization of CDPK and CRK genes

**Figure 3:**
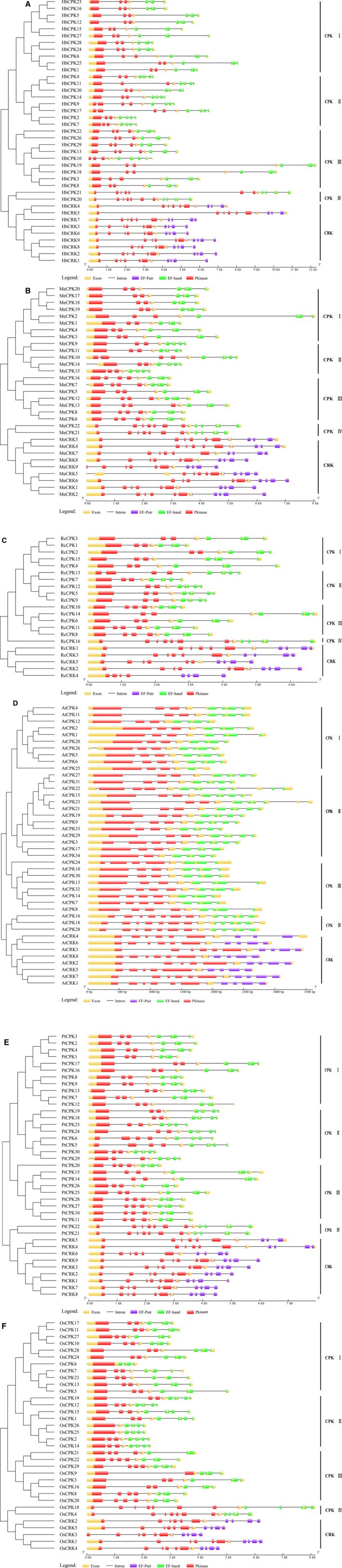
Structural organization of *CDPK* and *CRK* genes from *H. brasiliensis* and five other plant species. (A–F), structural organization of *CDPK* and *CRK* genes in *H. brasiliensis*,* M. esculenta*,* R. communis A. thaliana, P. trichocarpa*, and *O. sativa,* respectively. Exons and introns are represented by boxes and black lines, respectively. The Ser/Thr kinase catalytic domain is represented by red boxes. The EF‐hand domains predicted by Pfam and ProSiteProfiles are represented by boxes of green, whereas the EF‐pair domains predicted by Gene3D are represented by boxes of purple, which were degenerative Ca^2+^‐binding EF‐hands. The sizes of exons and introns are proportional to their sequence lengths.

The exon–intron structures of the 205 *CDPK* and *CRK* genes in six plant species were determined based on the predicted sequences. As shown in Fig. 3A, most *H. brasiliensis CDPK* members within the same groups share very similar gene structures in terms of intron number, domain localization, and exon length. Although the lengths vary, introns are inserted into nearly the same locations of the gene ORFs. There are 6, 7–8, 6–9, 10, and 11 introns in the CPK I, CPK II, CPK III, CPK IV, and CRK groups, respectively. The number of introns in different CPK and CRK groups is broadly similar in the five other plants examined (Fig. 3B–F), containing mainly 5–8 introns in CPK I to III groups, and 11–12 introns in CPK IV and CRK groups. The similar exon–intron structure shared by CPK IV and CRK genes reflects their close phylogenetic relationship. Most *CDPK* members contain two EF‐hand pairs, except for a few members, such as *AtCPK25*, which contains only one EF‐hand pair. It is noted that the EF‐pair domain in group CRK is a degenerative form of the EF‐hand domain that could be predicted with Gene3D but not with Pfam and ProSiteProfiles as used for the identification of the EF‐hand pairs in CPKs. In addition, the lengths of most *AtCPK* genes are shorter than those of the other plant *CDPK* genes, suggesting a correlation of the *CDPK* gene length with the genome size of its source species.

### Expression analysis of CDPK and CRK genes in six plant species

**Figure 4:**
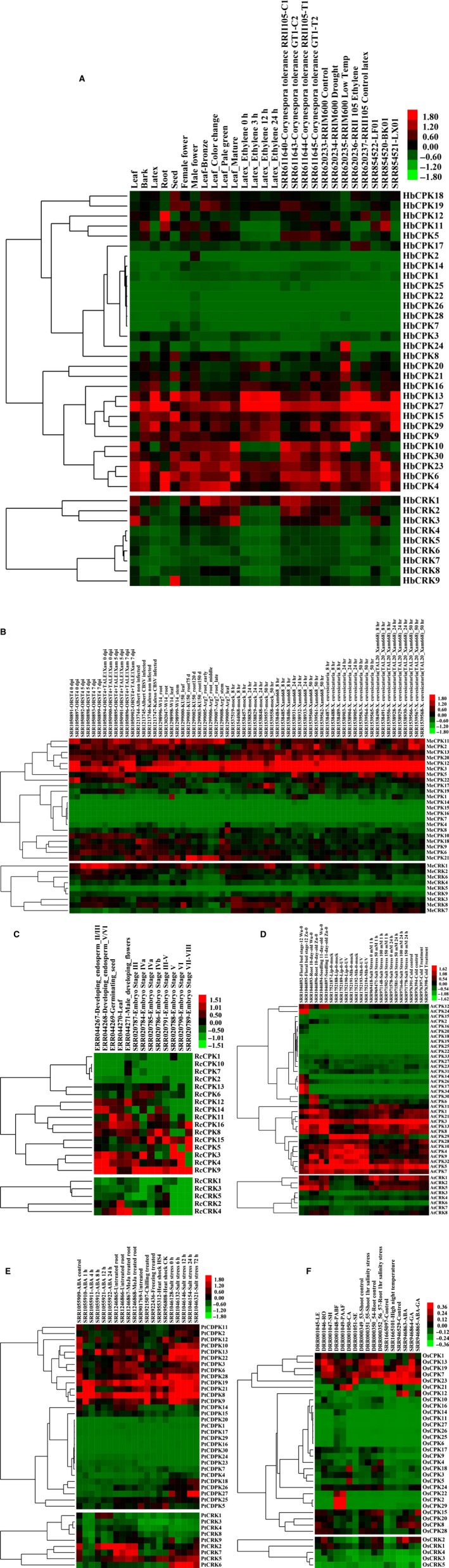
Expression analyses of the *CDPK* and *CRK* genes based on Solexa sequencing. (A), hierarchical clustering and differential expression analysis of the *HbCPK* and *HbCRK* genes in seven tissues (leaf, bark, latex, root, seed, female flower, male flower), at four developmental stages of leaves (bronze, color change, pale‐green, and mature), during ethephon treatment (0, 3, 12, and 24 h), *Corynespora cassiicola* tolerance (PRJNA179126), abiotic stress (drought and low temperature, PRJNA182078; and ethephon treatment, PRJNA182079), and tissues (leaf, bark, and latex, PRJNA201084); (B), hierarchical clustering and differential expression analysis of the *MeCPK* and *MeCRK* genes in different tissues (root, leaf, stem, PRJNA248260), infected by pathogenic Xanthomonas (PRJNA231851), CBSV virus (PRJNA243380), and bacterial blight pathogen (PRJNA257332); (C), hierarchical clustering and differential expression analysis of the *RcCPK* and *RcCRK* genes in different tissues (PRJEB2660) and during four oilseed development stages (PRJNA79463); (D), hierarchical clustering and differential expression analysis of the *AtCPK* and *AtCRK* genes in different tissues (floral bud, root, seeding, PRJNA231088), UV treatment (PRJNA272425), cold stress (PRJNA218632), salt stress (0, 50, 100, 150 mm, PRJNA217812); (E), hierarchical clustering and differential expression analysis of the *PtCDPK* and *PtCRK* genes under ABA stimulation (0, 1, 4, 8, 12, and 24 h, PRJNA232098), methyl jasmonate stimulation (PRJNA244820), chilling, freezing, and heat shock (PRJNA207974, PRJNA215888), salinity stress (0, 6, 12, 24, and 72 h, PRJNA230867); (F), hierarchical clustering and differential expression analysis of the *OsCPK* and *OsCRK* genes in different tissues (LE: leaf, RO: root, SH: shoot, PABF: panicle before flowering, PAAF: pannicle after flowering, CA: callus, SE: seed, PRJDA67119), salinity stress (PRJDA46487), high night temperature stress (PRJNA267031), abscisic acid (ABA), and gibberellic acid (GA) treatments (PRJNA213797).

Increasing evidence indicates that *CDPKs* are involved in various physiological adaptations 17, 19, 42. Such functions are indicated by the expression of *CDPK* genes in response to various stimuli, for example, hormones, salt, cold, drought, heat, and wounding. To understand the potential functions of specific members of *CDPK*s and *CRK*s in the six plant species, their expression patterns in different tissues, developmental stages, and under different stress treatments were analyzed. The Solexa sequencing data available at the NCBI Sequence Read Archive (SRA) database were used for the expression analysis of *CDPK* and *CRK* genes in five species, i.e. *M. esculenta, R. communis A. thaliana, P. trichocarpa*, and *O. sativa*. In the case of *H. brasiliensis*, two sets of Solexa sequencing data were used, one from the NCBI SRA database (http://www.ncbi.nlm.nih.gov/nuccore/448814761) and the other from our own data 38. As shown in Fig. 4A, transcripts of nine *HbCPK* genes (*HbCPK1, 2, 3, 7, 14, 17, 22, 25, 26*, and *28*) and five *HbCRK* genes (*HbCRK4, 5, 6, 7*, and *8*) were barely detectable in almost all the tissues and all the treatments examined. Such genes comprise a large portion (~ 1/3) of the total *HbCPK* and *HbCRK* gene family. This character seems to be shared by the *CDPK* and *CRK* gene families in other plant species. For example, similar expression patterns were observed for eight of 31 *CDPK* and *CRK* genes in *M. esculenta* (Fig. 4B), seven of 21 in *R. communis* (Fig. 4C), 15 of 42 in *A. thaliana* (Fig. 4D), 13 of 39 in *P. trichocarpa* (Fig. 4E), and 13 of 35 in *O. sativa* (Fig. 4F). This result suggests that the *CDPK* and *CRK* gene families in higher plants may have experienced an event of gene expansion followed by nonfunctionalization in the course of evolution 43. Due to the data limitation of the SRA database, some analyses may not be so accurate and need further verification. For example, *AtCPK16* and *AtCPK20* are highly expressed but only in pollen which has not been analyzed here 44. Among the expressed *H. brasiliensis* genes, *HbCPK24* and *HbCRK9* were special for their highly specific expression, which showed abundant expression in the leaf when exposed to low temperature and in the seed, respectively, but their expression were otherwise hardly detected in nearly all the tissues and treatments (Fig. 4A). In comparison, the remaining *HbCPK and HbCRK* genes were expressed in a wide range of tissues, showing dissimilar but partially overlapping patterns of expression. In the bark, where the rubber‐producing laticifers are located, 16 *HbCPK* genes were expressed more abundantly than in the other tissues (Table S1–1, Fig. 4A), and these genes may contain the key *CDPK* member s involved in signaling transduction in the rubber tree bark. *HbCPK13* and *27* were abundantly expressed in latex, essentially the cytoplasm of laticifers, whereas *HbCPK10* was mainly expressed in leaves (Table S1–1, Fig. 4A). To obtain further information on the *HbCPK* and *HbCRK* genes in leaf development, we examined their expression levels by RNA‐seq at four progressive stages of leaf development (bronze, color change, pale‐green, and mature). As shown in Fig. 4A and Table S1–1, most of the *HbCPK* and *HbCRK* genes were obviously down‐regulated during leaf development, and their expression was mainly restricted to the first three stages (bronze, color change, and pale‐green). This result is in agreement with the assay of CDPK activity in maize, in which expanding tissues such as rapidly growing tips, leaves, and coleoptiles have particularly high levels of CDPK activity, while mature leaves have much less activity 45. The involvement of *CDPK* genes in early developmental processes such as embryogenesis, seed development, and germination has been reported in sandalwood 46. Here, we reveal that one *R. communis CDPK* gene in subgroup CPK IIc, *RcCPK12*, was mainly expressed in the early stages of embryo development and the male developing flowers, suggesting a similar function. It is interesting that the other members of this subgroup were either expressed at a low level or they showed no expression.

Ethephon, an ethylene generator, is widely used in *H. brasiliensis* to stimulate rubber production, but the yield‐stimulating mechanisms are still poorly understood 29, 31. Ethylene‐mediated cross‐talk has been observed between CDPK and MAPK, indicative of signaling that controls stress responses in plants 23. In this study, we examined the expression levels of *HbCPK* and *HbCRK* genes in latex under ethephon treatment. As shown in Fig. 4A and Table S1–1, the expressions of two *CPK* genes, *HbCPK9* and *15*, were obviously up‐regulated by ethephon treatment, implicating them in the ethylene‐simulated latex production. Further studies on these genes would help to understand the underlying molecular mechanism. Although the expression of one *HbCPK* gene, *HbCDPK1* (*HbCPK30* in this study) has been reported to be markedly induced by ethephon 31, it was expressed mainly in the leaf and only marginally in latex in the present study (Fig. 4A). In *P. trichocarpa,* most of the *CDPK* and *CRK* genes were associated with ABA activity, but several genes were also affected by MeJa (methyl jasmonate), which apparently down‐regulated the expression of *PtCDPK1*,* 4*, and *11* (Fig. 4E, Table S1). In *O. sativa,* many *CDPK* genes were up‐regulated by treatment with ABA, GA (gibberellic acid), or ABA plus GA. In a previous study, *AtCPK4* and *AtCPK11* have been demonstrated to be involved in ABA signaling‐mediated regulation of seed germination, seedling growth, stomatal movement, and salt stress tolerance 17. Together, these results suggest that the *CDPK* and *CRK* gene families are implicated in the regulation of plant growth and development through participation in the signaling pathways of various plant hormones, especially ABA.

As low temperature is the major obstacle to the expansion of rubber planting areas in the subtropics, the studies of the low‐temperature response and adaptation in this species would be helpful in breeding rubber trees for cold tolerance. Recently, Ma *et al*. 47 found that low temperature sensing in rice was related to changes in Ca^2+^ influx during cold treatment, and the change in Ca^2+^ influx was controlled by COLD1, a regulator of G‐protein signaling. In this study, the expression levels of *HbCPK* and *HbCRK* genes (Ca^2+^ sensors) were examined under cold stress. Many *HbCPK* and *HbCRK* genes are regulated by cold stress, and most of them showed depressed expression after low‐temperature treatment (Fig. 4A, Table S1–1). In three of the other plants examined (*A. thaliana*,* O. sativa*, and *P. trichocarpa*), the expression of many *CDPK* and *CRK* genes was also regulated by low temperatures, but different expression patterns were displayed by respective genes (Fig. 4D–F; Table S1–4, 1–5 and 1–6). In addition, the expression data used in this study also show some discrepancies with other published studies. For example in rice, *OsCPK9* and *OsCPK21* have been reported to be induced by salt stress and ABA 22, 48, which is not observed in Fig. 4F. These differences may be due to different approaches, kinetics, or studied organs/ tissues.

**Figure 5:**
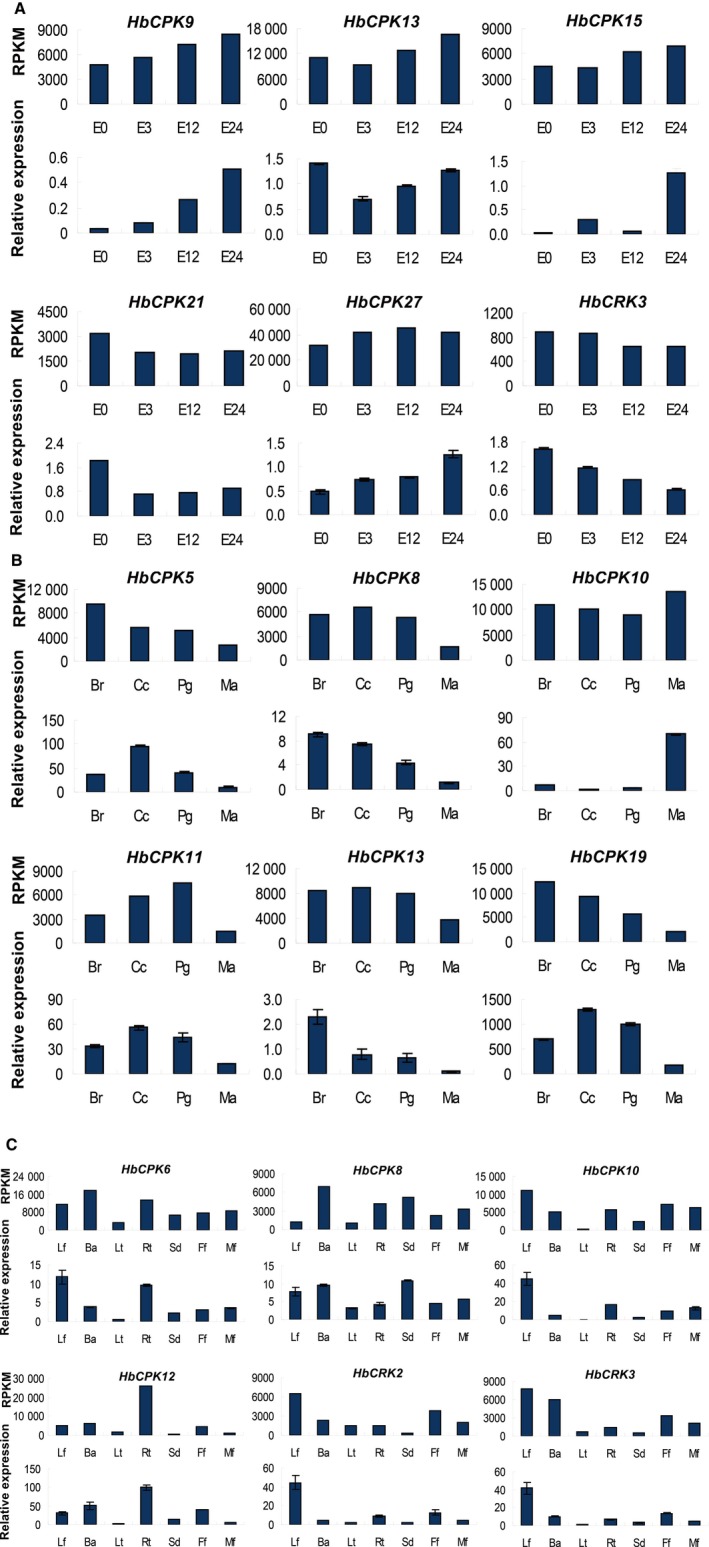
Expressional analyses of *HbCPK* and *HbCRK* genes by quantitative PCR and Solexa sequencing. (A) the expression patterns of six genes after ethephon treatment (0, 3, 12, and 24 h); (B) the expression patterns of six genes at four progressive stages of leaf development, that is, bronze (Br), color change (Cc), pale‐green (Pg), mature (Ma); C, the expression patterns of six genes in seven tissues, that is, leaf (Lf), bark (Ba), latex (Lt), root (Rt), seed (Sd), female flower (Ff), and male flower (Mf). For each gene, the RNA samples used for qPCR assays were the same as Solexa sequencing, the expression patterns were compared by using Solexa sequencing (upper panel) and quantitative PCR (lower panel). The results of quantitative PCR was shown as means ± STDEV of three technical replicates.

**Figure 6:**
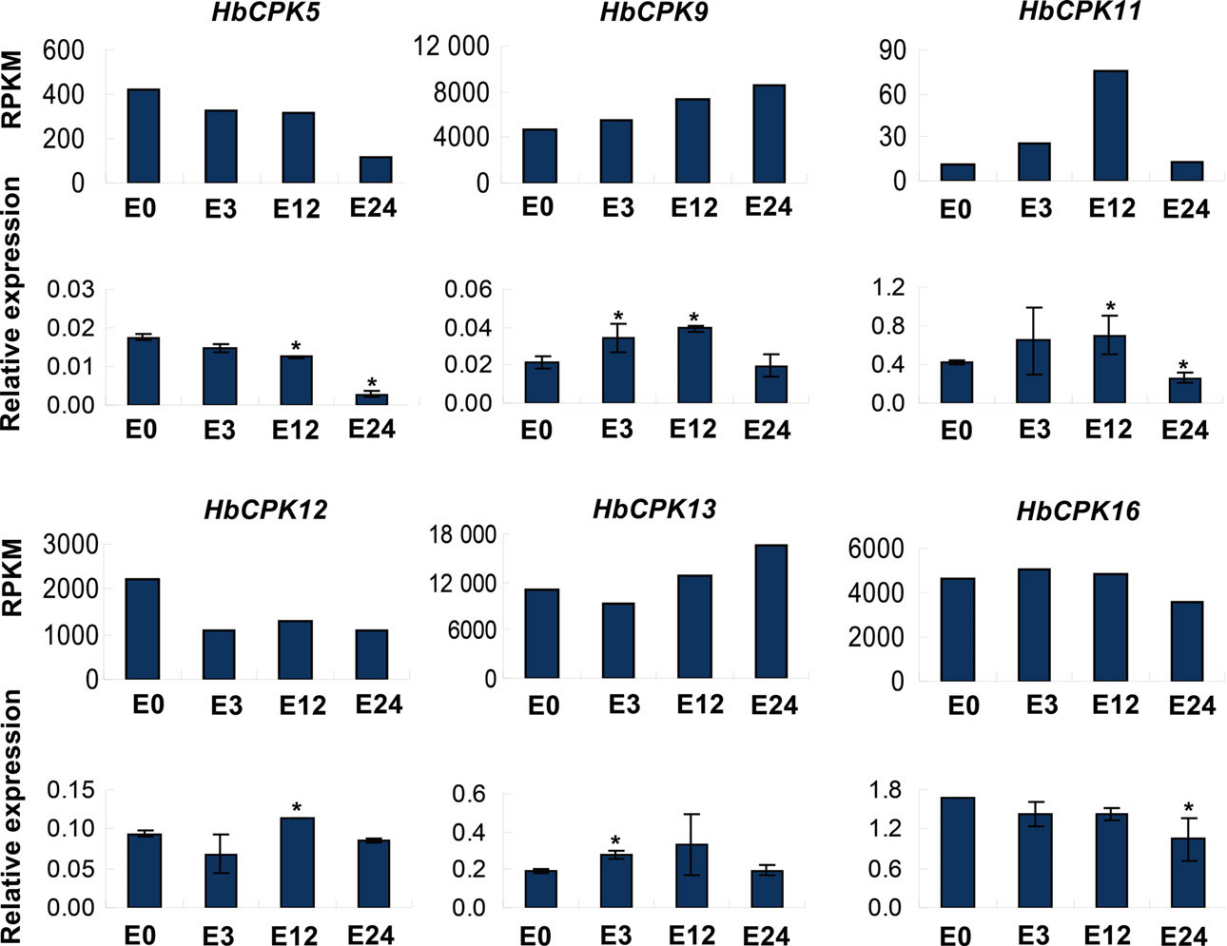
Expression of six *HbCPK* genes in response to ethephon treatment as assayed by qPCR and Solexa sequencing. The expression of six *HbCPK* genes in the latex was analyzed after different intervals (0, 3, 12 and 24 h) from ethephon treatment with new samples, which were different from the samples of Solexa sequencing and have three biological replicates. For each gene, the expression patterns from Solexa sequencing (upper panel) and by qPCR (lower panel) are compared. qPCR results are means (*n* = 15) ± STDEV of three biological replicates. Each time point was compared with 0 h. Asterisks indicate significant differences (Student's *t*‐test, *P* < 0.05).

The expression levels of *CDPK* and *CRK* genes in *H. brasiliensis*,* A. thaliana*,* O. sativa*, and *P. trichocarpa* were also examined under other kinds of stresses, including fungus infection, drought, and salt. The results showed that these treatments could also regulate the expression of many *CDPK* genes (Fig. 4A,D–F, Table S1). Under these stresses, the *H. brasiliensis* and *P. trichocarpa CDPK* and *CRK* genes were mainly down‐regulated, while the *A. thaliana* and *O. sativa* genes were mainly up‐regulated (Table S1). To verify the expression patterns of the RNA‐seq analyses, quantitative reverse transcriptase PCR (qPCR) expressional analyses were performed on *HbCPK* genes, which are among the isoforms somewhat regulated by ethephon treatment and leaf development, or tissue‐specific as revealed by RNA‐seq data (Fig. 4A; Table S1–1). First, qPCR expressional analyses were performed with the RNA samples from the same cultivated varieties as Solexa sequencing (our own data). As shown in Fig. 5, the results from sequencing‐based expression analyses were broadly similar to the qPCR results. Second, new RNA samples were used to further verify the expression patterns of *HbCPK* genes which are regulated by the ethephon treatment, the new RNA samples were different from the samples of Solexa sequencing and have three biological replicates. As shown in Fig. 6, the results from sequencing‐based expression analyses were broadly similar to the qPCR results at least one time point post treatment. The RNA‐seq data were also verified by our published analyses 38, 49. Above all, we can verify that the results of the RNA‐seq analyses on *HbCPK* genes are authentic.

### Expression analysis of CDPK and CRK genes based on structure and evolution

The two closely related genes in each of the *CDPK* and *CRK* paralogous gene pairs mentioned earlier may have similar cellular localization and similar roles, as reported for the *A. thaliana AtCPK4* and *AtCPK11* 17. However, when the expression patterns of the *H. brasiliensis* paralogous gene pairs were analyzed (Fig. 4A), the two genes in the same gene pair displayed four different expression patterns. First, in the case of three gene pairs (*HbCPK1/25, HbCPK2/7*, and *HbCPK22/26*), both genes were expressed either at a low level or not at all in all the tissues and treatments examined. Second, in the gene pair of *HbCPK5/12* and *HbCPK18/19*, both genes exhibited a similar pattern of universal expression across all tissues. Third, for the gene pair of *HbCRK3/6*, the two genes displayed dissimilar patterns of expression, with *HbCRK3* showing universal expression but *HbCRK6* being expressed at very low levels in all the tissues or treatments. Fourth, for the gene pair of *HbCPK24/28*,* HbCPK28* was barely expressed in all the tissues or treatments, but the expression of *HbCPK24* was only restricted in leaf when exposed to the low‐temperature treatment. We also investigated the expression patterns of the genes pairs or clusters that are closely located on chromosomes of *A. thaliana*,* O. sativa*, and *P. trichocarpa* (Fig. 2). The results showed that the two paralogous genes in most of the gene pairs shared similar expression except *AtCPK5* and *6* (Fig. 4D–F). *AtCPK5* was expressed at very high levels in all the samples examined, while *AtCPK6* was mainly specifically expressed in the root. In the paralogous gene cluster of *AtCPKs* in subgroup CPK IIe, only *AtCPK21* was expressed in all the tissues and treatments, whereas the other members (*AtCPK22, 23, 27,* and *31*) were expressed at low levels or not at all in all the samples examined. Interestingly, the *CDPK* and *CRK* genes from different plant species that exhibited low or no expressions in nearly all the samples examined tended to cluster in the same subgroups, for example, CPK Ib and c, CPK IIa and c, CPK IIIa and CRK a (Figs 1 and 4). This kind of phenomenon can be extended to the universally expressed *CDPK* and *CRK* genes from different species. These findings suggest that the structures of some *CDPK* and *CRK* gene families coevolved with their expression patterns in higher plants.

## Conclusion

In this study, we pioneered a genome‐wide analysis of two protein kinase subfamilies (CDPK and CRK) in *H. brasiliensis*,* M. esculenta*, and *R. communis*. *In silico* analysis of the *H. brasiliensis* genome database allowed the identification of 30 *HbCPK* and 9 *HbCRK* genes. The phylogenetic analysis of CDPKs and CRKs from *H. brasiliensis* and five other plant species (*A. thaliana, O. sativa, P. trichocarpa, M. esculenta*, and *R. communis*) showed the classification of these genes into five major groups. Members within each group might have recent common evolutionary origins, as they share common protein motifs and exon–intron structures. Solexa sequencing analyses revealed that most *H. brasiliensis CDPK* and *CRK* genes exhibit different patterns of expression under a number of experiments, suggesting their distinct roles in developmental and stress responses. Relevant studies of gene evolution and expression have been extended to the five other plant species. The results presented here provide a foundation for further functional investigation of the *CDPK* and *CRK* gene families in *H. brasiliensis* as well as the whole plant kingdom.

## Materials and methods

### Database search for CDPK and CRK genes in *H. brasiliensis* and five other plant species

Sequences of *A. thaliana*,* P. trichocarpa*, and *O. sativa CDPK*s and their closely related *CRK* genes were downloaded from the *A. thaliana* Information Resource (http://www.Arabidopsis.org/), the rice genome annotation database (http://rice.plantbiology.msu.edu/) and GenBank (http://www.ncbi.nlm.nih.gov/genbank), respectively. The genome and protein sequences of *A. thaliana*,* O. sativa*,* P. trichocarpa*,* M. esculenta*, and *R. communis* were downloaded from Phytozome v10 (http://www.phytozome.net/). The *H. brasiliensis* genome and transcriptome data were obtained from GenBank (http://www.ncbi.nlm.nih.gov/nuccore/448814761) and our own data 38. Local BLAST alignment was performed using published *CDPK* and *CRK* sequences form *A. thaliana*,* P. trichocarpa*, and *O. sativa* as queries to search against the deduced proteome of each species for the candidate *CDPK*s and *CRK*s from *H. brasiliensis*,* A. thaliana, O. sativa, P. trichocarpa, M. esculenta*, and *R. communis*. All putative candidates were manually verified with the InterProScan server (http://www.ebi.ac.uk/Tools/pfa/iprscan/) to confirm the presence of protein kinase and EF‐hand domains.

### Phylogenetic and gene structure analyses

Multiple alignments of the amino acid sequences of CDPKs and CRKs from six species were performed using the clustal x (version 1.83, http://www.clustal.org/) program. The phylogenetic tree was constructed with mega6.0 (http://www.megasoftware.net/) by employing the Neighbor‐Joining (NJ) method with a bootstrap test for 1000 replicates. Exon–intron structures of the six species of *CDPK* and *CRK* genes were analyzed by comparing the cDNA and their genomic DNA sequences through the web server gsds 2.0 (http://gsds.cbi.pku.edu.cn/).

### Expression analysis based on Solexa sequencing

For Solexa sequencing‐based expression analyses, SRA data were downloaded from the NCBI database. The sequences included those for *H. brasiliensis* (*Corynespora cassiicola* tolerance, PRJNA179126; abiotic stress, PRJNA182078 and PRJNA182079; tissues, PRJNA201084; tissues, leaf development, and ethephon treatment 38), *M. esculenta* (*Xanthomonas* tolerance, PRJNA231851; CBSV virus infected, PRJNA243380; tissue, PRJNA248260; bacterial blight pathogen infected, PRJNA257332), *R. communis* (tissue, PRJEB2660; developing oilseeds, PRJNA79463), *A. thaliana* (salt stress, PRJNA217812; tissues, PRJNA231088; UV treatment, PRJNA272425; cold stress, PRJNA218632), *O. sativa* (high night temperature stress, PRJNA267031; salinity stress, PRJDA46487; tissues, PRJDA67119; ABA and giberellic acid treatments, PRJNA213797), and *P. trichocarpa* (ABA stimulation, PRJNA232098; methyl jasmonate treatment, PRJNA244820; chilling, freezing and heat shock, PRJNA207974 PRJNA215888; salinity stress, PRJNA230867) (Table S2). For *H. brasiliensis* samples, additional datasets were used for expression analysis as described previously 38, 49. These sets included data for seven different tissues (latex, bark, leaf, root, seed, female flower, and male flower), leaves of four developmental stages (bronze, color change, pale‐green, and mature), and latex collected at 0, 3, 12, and 24 h after ethephon stimulation.

### Quantitative reverse transcriptase PCR (qPCR)

To verify the data obtained by Solexa sequencing, quantitative RT‐PCR (qPCR) was performed as describe previously 32. The RNA samples used for qPCR assays were the same as described previously 49. The reaction was conducted on the Light Cycler 2.0 system (Roche Diagnostics, Mannheim, Germany) by using the SYBR‐Green premix kit (TaKaRa) according to the manufacturer's protocol. The primer pairs used for the *HbCPK* and *HbCRK* genes were 5′‐AGATAGAGAGGGTCAACTCTG GAC‐3′ (F) and 5′‐GCTTGTCTTTGTTTTTTGTATGTG‐3′ (R) (*HbCPK5*), 5′‐TAGTTTCAGCATTG GATTTAGAGA‐3′ (F) and 5′‐AGTTATAAAGGTCTGCCTGCTTCT‐3′ (R) (*HbCPK6*), 5′‐TTCTTCC CTTTTGTTTTTTCTCTC‐3′ (F) and 5′‐CTTCTTCTTGCGTCTGTGATTTGT‐3′ (R) (*HbCPK8*), 5′‐C AAGTTCCCAACATTTAACCTTCT‐3′ (F) and 5′‐GAAGAGTCAGCGGCGTTAGC‐3′ (R) (*HbCP K9*), 5′‐AGAGAAAGATTCAATAGTCTGAGCT‐3′ (F) and 5′‐ACTTGTGATGTATTTACAACCACA TA‐3′ (R) (*HbCPK10*), 5′‐GGGTATCATCTGAATCTGCTTCTG‐3′ (F) and 5′‐GCAGTATCATTGTA GAGCCGTGG‐3′ (R) (*HbCPK11*), 5′‐CCATTAACACAAGCAGGTCAAGTC‐3′ (F) and 5′‐CGGTA AGGAAGAACCCA TTTG‐3′ (R) (*HbCPK12*), 5′‐GAT GGGTCATTAGAACTGAACAGT‐3′ (F) and 5′‐AACCAGGGCTCACATCCTATTTA‐3′ (R) (*HbCPK13*), 5′‐CTGCAACTCACAGATCATTCCCT C‐3′ (F) and 5′‐AAAGTTTTGCTCACGGCCTTC‐3′ (R) (*HbCPK15*), 5′‐ACCAACCACTGCTACCA ACG‐3′ (F) and 5′‐TACTTCCAAGACTGAGCAAAAGAT‐3′ (R) (*HbCPK16*), 5′‐CATTCAACGAT GAGGAAGACGAG‐3′ (F) and 5′‐TTTTTAAAGTGCAAATTTCGTCCC‐3′ (R) (*HbCPK19*), 5′‐GG AGACAAGTGAGAGAGTTACAGAA‐3′ (F) and 5′‐CATCCTATGGGGAATCCTACC‐3′ (R) (*HbCP K21*), 5′‐ATTCTTTCAGGTAAGCATGTATGC‐3′ (F) and 5′‐AGAAATTAAAAAGGGTTGTAAT CC‐3′ (R) (*HbCPK27*), 5′‐GGCACACTGATGGAAAGTTGAGT‐3′ (F) and 5′‐TTTTGGAGCTTCTG CATTAGTTTA‐3′ (R) (*HbCRK2*), and 5′‐CTGTGAAGAGTGCTGCAAGTTAAA‐3′ (F) and 5′‐GG TAAGAACAAAGAGGAAAAACAT‐3′ (R) (*HbCRK3*). The *H. brasiliensis UBC2b* and *UBC4* gene were used as internal controls as described previously 50. The details for experimental manipulations and data analysis were as described by Tang *et al*. 32.

## Author contributions

CRT conceived and designed the experiments; XHX, MY, JLS, and JYQ performed the experiments; XHX, MY, and YJF analyzed the data; XHX, MY, SNH, and CRT wrote the paper. All authors read and approved the final manuscript.

## Supporting information


**Table S1.** Characteristics of *CDPK* and *CRK* genes in six plant species (*H. brasiliensis, A. thaliana, P. trichocarpa,* and *O. sativa*).

**Table S2.** Basic information for the Solexa sequencing data of *Hevea brasiliensis* and five other plant species.
